# Impact of COVID-19 on online grocery shopping discussion and behavior reflected from Google Trends and geotagged tweets

**DOI:** 10.1007/s43762-023-00083-0

**Published:** 2023-02-22

**Authors:** Nemin Wu, Lan Mu

**Affiliations:** Department of Geography, University of Georgia, Athens, GA USA

**Keywords:** Twitter, Online grocery shopping, Google Trends, COVID-19, Panic shopping, Pandemic fatigue

## Abstract

People express opinions, make connections, and disseminate information on social media platforms. We considered grocery-related tweets as a proxy for grocery shopping behaviors or intentions. We collected data from January 2019 to January 2022, representing three typical times of the normal period before the COVID-19 pandemic, the outbreak period, and the widespread period. We obtained grocery-related geotagged tweets using a search term index based on the top 10 grocery chains in the US and compiled Google Trends online grocery shopping data. We performed a topic modeling analysis using the Latent Dirichlet Allocation (LDA), and verified that most of the collected tweets were related to grocery-shopping demands or experiences. Temporal and geographical analyses were applied to investigate when and where people talked more about groceries, and how COVID-19 affected them. The results show that the pandemic has been gradually changing people’s daily shopping concerns and behaviors, which have become more spread throughout the week since the pandemic began. Under the causal impact of COVID-19, people first experienced panic buying groceries followed by pandemic fatigue a year later. The normalized tweet counts show a decrease of 40% since the pandemic began, and the negative causal effect can be considered statistically significant (*p*-value = 0.001). The variation in the quantity of grocery-related tweets also reflects geographic diversity in grocery concerns. We found that people in non-farm areas with less population and relatively lower levels of educational attainment tend to act more sensitively to the evolution of the pandemic. Utilizing the COVID-19 death cases and consumer price index (CPI) for food at home as background information, we proposed an understanding of the pandemic’s impact on online grocery shopping by assembling, geovisualizing, and analyzing the evolution of online grocery behaviors and discussion on social media before and during the pandemic.

## Introduction

More than a quarter of the global population has shopped online at least once (eMarketer, 2022). When the COVID-19 pandemic swept the world in 2020, pandemic-related stress, anxiety, and grief resulting from the more than 960,000 COVID deaths in the United States (US) had a visible impact on lifestyle and behavioral changes. People stayed at home, kept socially distant from others, and many were not shopping as frequently as they normally would (Statista, 2021a; Zamboni et al., 2021). The virus was considered a public health emergency in March 2020. Following the next 11 months, over 70% of consumers in the US claimed that the pandemic impacted their usual shopping behaviors (Numerator, 2021). While the crisis caused a decline in physical store shopping, it boosted online shopping (Baarsma & Groenewegen, 2021). Restaurant closures, quarantine policies, and work-from-home lifestyles during the pandemic affected dietary choices and online grocery shopping behaviors especially. According to a report published in March 2020, 42% of the US population purchased their groceries online at least once a week, marking a stark increase from 22% just 2 years ago (GeekWire, 2020).

Right after the coronavirus was considered a public health emergency, the pandemic triggered panic-buying. Panicked shoppers emptied shelves due to anxiety, leading to stockouts and supply chain disruptions (Yuen et al., 2020). Many consumers chose to keep extra goods on hand after the shortages experienced at the beginning of the pandemic (Numerator, 2021). However, after 2 years into the pandemic, living with the daily noise surrounding COVID-19 in many streams of information 24/7, it is reasonable to assume no one has escaped feeling some degree of pandemic fatigue and tired of all the struggles it brought on (Giles, 2022).

Online shopping at major eCommerce sites such as Amazon and Walmart is related to the use of Twitter and other social media (Vithayathil et al., 2020). However, the impact of the COVID-19 pandemic on online grocery shopping behavior and buying tendencies, as evidenced through social media data, has not been extensively studied. Twitter is a popular social media platform for people to express opinions, make connections, and disseminate information (Broersma & Graham, 2013). This paper considers grocery-related tweets as a proxy for grocery shopping behaviors or intentions. Tweets are publicly available data that provide some levels of geoinformation when users choose to tag their current location in their tweets. Obtaining geotagged tweets allows us to specify the spatial variations among people’s reactions to COVID-19.

With the grocery-related online discussion and shopping behavior data, we want to answer the following questions:

1. Has the pandemic been changing people’s daily grocery-related shopping behaviors and online discussions, and how?
2. Are there geospatial and temporal variations in grocery-related concerns?
3. How to understand the COVID-19 pandemic’s impact on the evolution of online grocery behaviors and discussions on social media before and during the pandemic?

## Materials and methods

### Data

We began by collecting geotagged tweets through the Twitter API v2 for Academic Research using a keyword query over 3 years, from 01/20/2019 to 01/19/2022. Since the US food & grocery retail market is dominated by chain supermarkets, the keywords we selected contain the names of the top 10 leading grocery chains in the US (e.g., Walmart, Kroger, and Costco) to match grocery-related tweets (SN Supermarket News, 2021; Statista, 2021b). January 20 was chosen as the start date since the CDC confirmed the first US laboratory-confirmed case of COVID-19 on January 20, 2020. Phase I of data acquisition lasted from February 20, 2019, to February 19, 2020 (before the COVID pandemic spread in the US). Phase II lasted from February 20, 2020, to February 19, 2021 (the outbreak period of COVID-19). Phase III lasted from February 20, 2021, to February 19, 2022 (the widespread period of the pandemic). In this study, we selected only tweets that contained geospatial information in the US to analyze their spatial distribution. Although the counts of retweets indicate the impact of the original tweets, retweets are only re-sending existing tweets. We only collected original tweets, which offer a more personal perspective and interpretation on grocery shopping, as well as quote tweets, which reference existing tweets with added comments. We limited the language to English and specified the geographic extent to US to ensure a consistent language and context environment for data collection and processing. Tweets posted from the official accounts (e.g., @WalmartInc, @KrogerNews, @Costco, @costco_USA, etc.) were also removed because they could not represent the consumers’ perspectives.

Data about online grocery shopping behavior was gathered at the state level from grocery-related Google Shopping searches on Google. Values represent search interest in grocery-related Google Shopping for the given region and time.

### Methods

#### Spatial analysis

Fewer and fewer Twitter users choose to share their verified location in tweets (Huang & Carley, 2019). According to a large-scale empirical study of geotagging behavior on Twitter in 2019, 1.76% of tweets have geoinformation, while only 0.55% have precise coordinates (Huang & Carley, 2019). The amount of grocery-related tweets with accurate coordinates is too limited. To mitigate the insufficiency of precise coordinates yet still maintain valid location information for spatial analysis, we included tweets with bounding boxes (Etienne et al., 2019). However, the bounding box can range from a venue to an entire region (Census, 2020), so it is necessary to filter the tweets with small bounding boxes and exclude those with big boxes. We set a threshold to filter the size of the bounding box at the state level, taking the centroid of the bounding box as the coordinates where the tweet is posted. We decide on a circle with the average land area of a US state and set the threshold of the bounding box’s side length with the radius of that circle.

To study the change in people’s concern about groceries at a local level, we used an allocation gridding algorithm to assign tweet counts to 2.5 arcminutes (about 5 km at the equator, and 3 to 4 km for most places in the US) grid cells. For each grid, we calculated the difference in the total number of tweets between the current year and the previous year. To better understand the local changes, we selected the top three most populous metropolitan statistical areas, New York-Newark-Jersey City, Los Angeles-Long Beach-Anaheim, and Chicago-Naperville-Elgin, enumerated by the 2020 US Census. A classified symbology scheme is used to distinguish between high and low values.

We also aggregated the tweets to the state level to analyze the geographical variance across the country to better highlight and compare the pandemic panic and pandemic fatigue. We used quantile classification to divide the states into three categories by their annual rates of tweet count change. Two choropleth maps were created to visualize how pandemic panic and pandemic fatigue varied across the US. We further calculated the Pearson correlation coefficient (Steiger, 1980) between the annual rates of tweet count change and land use & demographics by state to examine the potential linear relationships.

#### Temporal analysis

The dataset returned from Twitter only includes an iso-1861 format time when the tweet was created. We used Bing Map API as well as the datetime and pytz packages in Python to determine the time zone of each tweet by its coordinate generated in the previous step, thus transferring the iso-1861 format time to the local time.

We aggregated the total counts of hourly tweets in a week for 3 years. For each time span, we compiled the frequency score into 7 (days of a week) by 12 (months) grids and created a heat map of tweets on groceries.

#### Topic modeling

We built a Latent Dirichlet Allocation (LDA) model for text classification to understand the content of the collected tweets. The LDA model is the best-known unsupervised learning technique for topic modeling (Suominen & Toivanen, 2016). We treated each tweet of a user as a single document. LDA considers each topic as a collection of keywords in a certain proportion. If the model knows the word frequency and which words often appear in the same document, it will discover patterns that can group different words together. Once the researcher provides the model with the number of topics, the LDA algorithm will find a user-selected number of topics within a specific corpus and learn the distributions of topics by rearranging the distribution of the keywords within the topics until obtaining a good composition of the topic-keywords distribution (Blei, 2003).

To prepare the clean tweets to build an LDA model, we removed URLs and usernames from the texts, tokenized each tweet, lowercase all words, lemmatized words, and removed stop words, punctuations and remaining special characters sequentially. We used the pyLDAvis package (Sievert and Shirley, 2014) in Python to build the LDA model for counting words and grouping similar word patterns to describe topics within the grocery-related tweets.

Topic coherence score evaluates the topic by measuring the degree of semantic similarity between high scoring words in it. A good model will generate topics with high topic coherence scores. While fine-tuning the model, we tried the number of topics (k) from 3 to 20 and iteratively computed the topic coherence scores. We determined the number of topics for the final LDA model with the highest average coherence score (Mimno et al., 2011).

#### Causal impact analysis

To validate the causal impact of COVID-19 on grocery-related Twitter discussions, we performed a causal inference using counterfactual predictions based on the Bayesian Structural Time Series (BSTS) model (Numerator, 2021). BSTS model was initially proposed by Google to infer the causal impact of market intervention. It improves on existing techniques (Danaher & Rust, 1996; Seggie et al., 2007; Stewart, 2009) in two aspects: first, it utilizes a robust series of Bayesian calculations along with predictor data sets to estimate the effect; second, it applies model averaging to build an adequate synthetic control for calculating the counterfactual (Brodersen et al., 2015). Specifically, BSTS provides a fully Bayesian time-series to predict a counterfactual time-series that would have occurred without the intervention. To assess whether the intervention significantly impacts the outcomes, BSTS then models the difference between the real outcome line with the invention and the estimated trend line without the invention (Brodersen et al., 2015). BSTS has been recently used in numerous studies to measure the impacts of the COVID-19 pandemic in various fields such as the public bicycle share system (Li et al., 2021), the stock market (Feng & Li, 2022) and mental health calls (Koziarski, 2021).

In our study, we wanted to estimate what the grocery-related tweet count would have been if they were not influenced directly by COVID-19. This counterfactual of time series is predicted by drawing upon two input datasets: the grocery-related tweet counts before and after COVID-19 appeared and the contemporaneous covariate that was not affected by the intervention. There are two core assumptions in BSTS to help determine the appropriate covariate: first, the covariate should be unaffected by the effects of treatment. When doing a causal impact study, we need to choose a control group that was hardly impacted by the intervention. The second criterion is that throughout the pre-period and the post-period, the relationship between the control group and the experiment group should remain constant (Brodersen et al., 2015). We took the weather-related tweet counts on Twitter as the control group because it satisfied the two selection criteria. The weather-related discussions have a stable relationship with the grocery-related Twitter discussions. The two phenomena’s confounding variables, such as the variation of Twitter user counts and the change of geotagged tweet counts due to the Twitter geotagging policy, have the same effect on both the experimental and control groups. Furthermore, weather-related tweets have hardly been affected by the intervention of the pandemic since people talk about the weather all the time, with or without COVID-19. The monthly count of grocery-related tweets is the treated series, and the monthly count of weather-related tweets is the control series. The intervention was added to the treated series when the first case of COVID-19 was reported in the US.

The Twitter data could be considered as people’s concerns and discussions about groceries. To observe the correlation between people’s thoughts and behaviors, we used people’s interest in searching for grocery shopping online from Google Shopping Trends as a proxy of online grocery shopping behaviors. We also used the daily increase in COVID-19 death cases to describe the development and severity of the coronavirus. The consumer price index (CPI) for food at home’s monthly growth rate indicates whether the economy is experiencing inflation, deflation, or stagflation over time. To keep the data consistent and facilitate comparison, all four variables, including tweet counts, Google Shopping Trends index, daily increase of COVID-19 death cases, and monthly CPI growth rate were normalized from 0 to 100 consolidated by month. All curves have been smoothed by the spline methods (Reinsch, 1967), *λ* is the smoothing parameter controlling the roughness of the function estimate. As *λ* → 0, there is no smoothing, and the smoothing spline converges to the interpolating spline. As *λ* → ∞, there’s infinite smoothing, and the estimate converges to linear least squares estimate. Based on the trade-off between fidelity to the data and the roughness of curves, the *λ* was set to be 0.5.(Kimeldorf & Wahba, 1970).

## Results

We collected 673,720 geotagged grocery-related tweets, of which 572,311 (84.95%) have bounding boxes that meet both thresholds we set for the state level. We located the filtered tweets to the centroids of their bounding boxes and generated the coordinates. The total count of grocery-related geotagged tweets in the US is 219,274, 222,917, and 130,120 for the three time spans.

### Spatial pattern

**Fig. 1:**
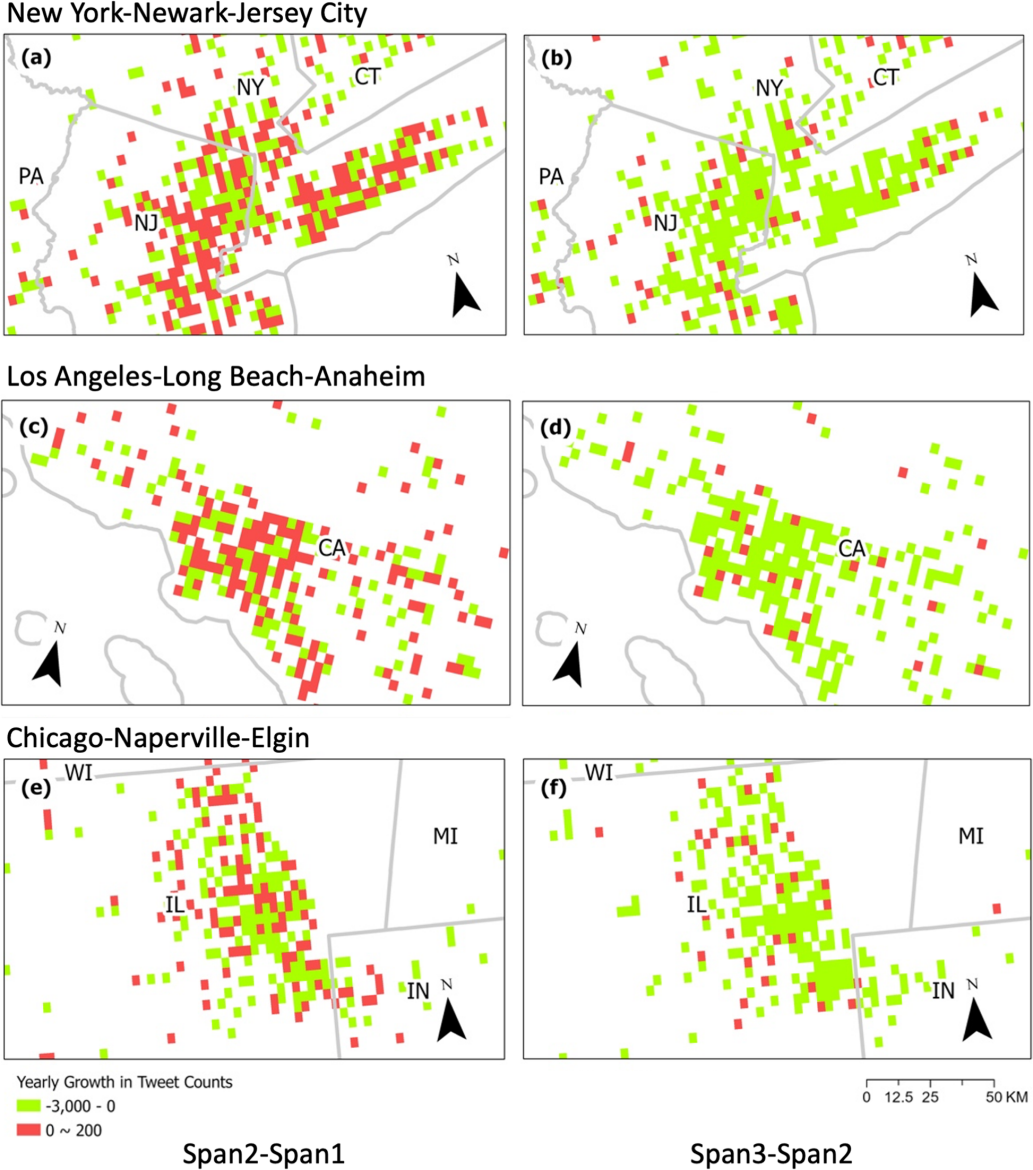
Annual change in tweet counts in metropolitan areas

Compared to the first time span (2/20/2019-2/19/2020, span1), the number of grocery-related tweets has slightly increased in the second time span (2/20/2020-2/19/2021, span2). The annual changes in the number of tweet discussions in the top three populous metropolitan areas are shown in Fig. 1. On Fig. 1, each row maps a top populous MSA, the first column shows the difference between spans 2 and 1, and the second column is for spans 3 and 2. The difference in tweet counts in an allocation grid is colored red for an increase and green for a decrease compared to the prior time span. This trend is noticeable in New York-Newark-Jersey City and Los Angeles-Long Beach-Anaheim (Fig. 1a and c). People cared about and talked more about grocery shopping on Twitter during the span2 (the first year of the COVID-19 pandemic). The pandemic triggered runs on grocery stores and panic-buying. Panicked shoppers emptied shelves due to coronavirus anxiety at the beginning of the pandemic (Giles, 2022).

However, there was a noteworthy phenomenon when it came to the third time span (2/20/2021-2/19/2022, span3), one year after the COVID pandemic started spreading in the US. In the entire country, including all three of the most populous MSAs (Fig. 1b, d and f), the number of green grids overwhelmingly exceeds the number of red grids, illustrating that people discussed less and did not mention groceries as much as last year. In response to the prolonged public health crisis, the public may feel demotivated and exhausted after months of spending extra time and energy dealing with meeting the demand of the new pandemic lifestyle (WHO, 2020).

**Fig. 2:**
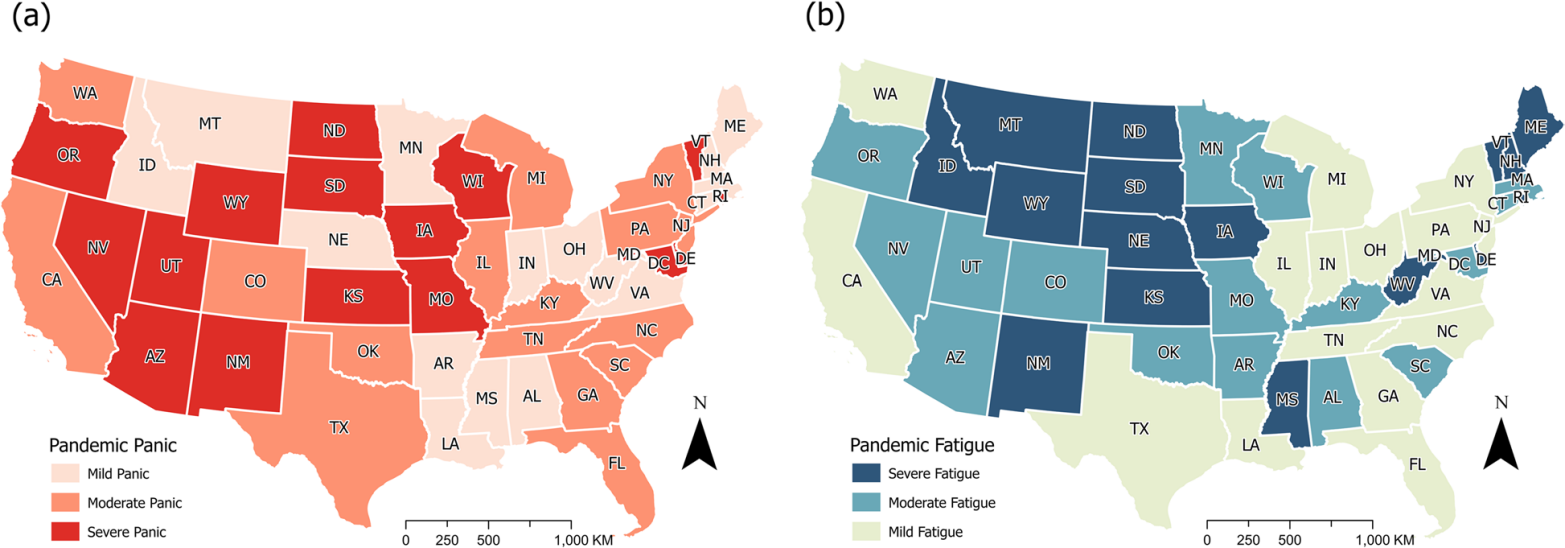
Pandemic panic (**a**) and pandemic fatigue (**b**) by state

The degree of increment in grocery-related tweet discussion during span2 reflects the phenomenon of pandemic panic and the public’s concern about accessing food resources. The degree of decrease during span3 concurs with the observed pandemic fatigue. In Fig. 2, two choropleth maps were created to further visualize the spatial variations of the pandemic panic and pandemic fatigue at the state level. Figure 2a describes the pandemic’s panic degree, calculated as the yearly rate of change in the number of tweets between span2 and span1. Figure 2b describes the pandemic’s fatigue degree, calculated as the yearly rate of change in the number of tweets between span3 and span2. The states are divided into three categories in both maps, each of which is assigned a distinct color from a graduated color scheme based on their yearly rates of change in tweet counts.

In Fig. 2a, the darkest and lightest reds indicate that the states showed the strongest and mildest pandemic panic.

- States demonstrated severe pandemic panic: AZ, DC, DE, IA, KS, MO, ND, NM, NV, OR, RI, SD, UT, VT, WI, WY
- States demonstrated moderate pandemic panic: CA, CO, FL, GA, IL, KY, MI, NC, NJ, NY, OK, PA, SC, TN, TX, WA
- States demonstrated mild pandemic panic: AL, AR, CT, ID, IN, LA, MA, ME, MN, MS, MT, NE, NH, OH, VA, WV

In Fig. 2b, the darkest and lightest blues indicate that the states showed the strongest and mildest pandemic fatigue.

- States demonstrated severe pandemic fatigue: DE, IA, ID, KS, ME, MS, MT, ND, NE, NH, NM, RI, SD, VT, WV, WY
- States demonstrated moderate pandemic fatigue: AL, AR, AZ, CO, CT, DC, KY, MA, MN, MO, NV, OK, OR, SC, UT, WI
- States demonstrated mild pandemic fatigue: CA, FL, GA, IL, IN, LA, NC, NC, NJ, NY, OH, PA, TN, TX, VA, WA

According to the results of Pearson’s r (Steiger, 1980), the total state population had a negative correlation coefficient of − 0.26 between the pandemic panic rate at a confidence level of 95%, and a negative correlation coefficient of − 0.47 between the pandemic fatigue rate at a confidence level of 99.9%. From Fig. 2, we can observe that California, Texas, Florida, New York, and Pennsylvania, the most populous states, appeared to be less vulnerable to both pandemic panic and panic fatigue. By contrast, states with small populations, such as Wyoming, Vermont, and North Dakota, appeared particularly sensitive to pandemic anxiety and panic fatigue. We also found that the pandemic panic rate was significantly correlated (*p*-value < 0.1) with the percentage of the population engaged in farming, fishing, and forestry occupations, with a correlation coefficient of − 0.19. The covid fatigue rate was also significantly correlated. The better educated population showed fewer signs of fatigue during the prolonged public health crisis since the pandemic fatigue rate was negatively correlated with a correlation coefficient of − 0.11 (*p*-value < 0.5) to the percentage of the population with a bachelor’s degree or higher. In a nutshell, we found that people in non-farm areas with less population and relatively lower levels of educational attainment tend to act more sensitively to the evolution of the pandemic.

### Temporal pattern

**Fig. 3:**
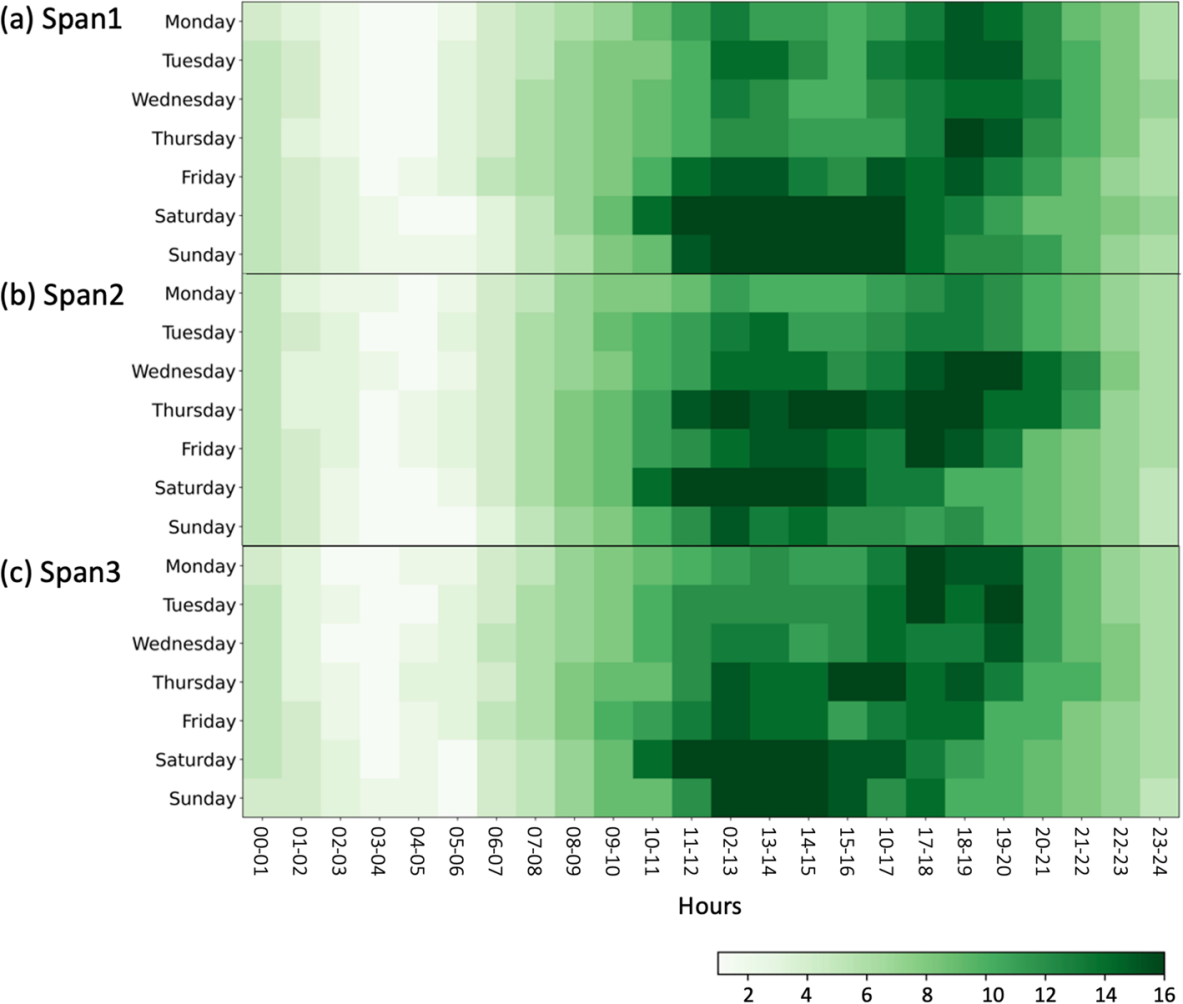
Twitter heat map about grocery

We discovered significant differences in tweet frequency over a week. In Fig.3, the grid with a darker color indicates a higher volume of discussions on grocery-related topics during that hour of the week. People’s attention and concerns about groceries on Twitter were more concentrated on the weekends during span1, a year before the outbreak. They primarily discussed groceries from 11 AM to 5 PM on weekends (Fig. 3a). In span2, the most popular time slots to discuss groceries were distributed more from Wednesday to Sunday (Fig. 3b). In span3, people even began to tweet a lot about groceries on Monday and Tuesday (Fig. 3c).

### Topic modeling

We found that the LDA performed the best when the number of topics was six with an average coherence score of 0.53. We inferred the name of topics by looking at all 30 relevant keywords and putting them into context of the tweets. The top-10 keywords for each topic were displayed in Table 1. The hottest topic about grocery-related tweets was about shopping demand. The topic ranked second was food-related, and chicken, pizza, and cheese were the three most popular foods to discuss on Twitter. People also liked to post their shopping experiences on social media, and most top keywords in topic 3 were positive. In sum, most of the tweets we collected were related to grocery-shopping demand or experience.

**Table 1:** The results of topics and their top-10 corresponding keywords from the 6-topic LDA model, along with the tweet counts under each topic

	Topics	Top 10 Keywords	Tweet Counts
1	Shopping demand	supercenter, need, store, food, shop, stop, today, new, day, buy	137,304 (24.0%)
2	Food and food preference	food, chicken, like, good, pizza, cheese, eat, buy, hot, grocery	116,299 (20.3%)
3	Shopping experience 1	shop, love, new, today, day, thank, great, see, local, food	95,871 (16.8%)
4	Shopping experience 2	food, open, come, post, know, photo, page, path, shop, pm	77,106 (13.5%)
5	COVID-19	people, store, like, get, mask, covid, employee, see, pay, will	59,910 (10.5%)
6	Time	get, time, go, today, lot, back, like, line, parking, right	43,169 (7.5%)
7	Related activity	supercenter, gasoline, gas, sign, city, north, today, close, paper, neighborhood	42,652 (7.5%)

### Causal impact analysis

**Fig. 4:**
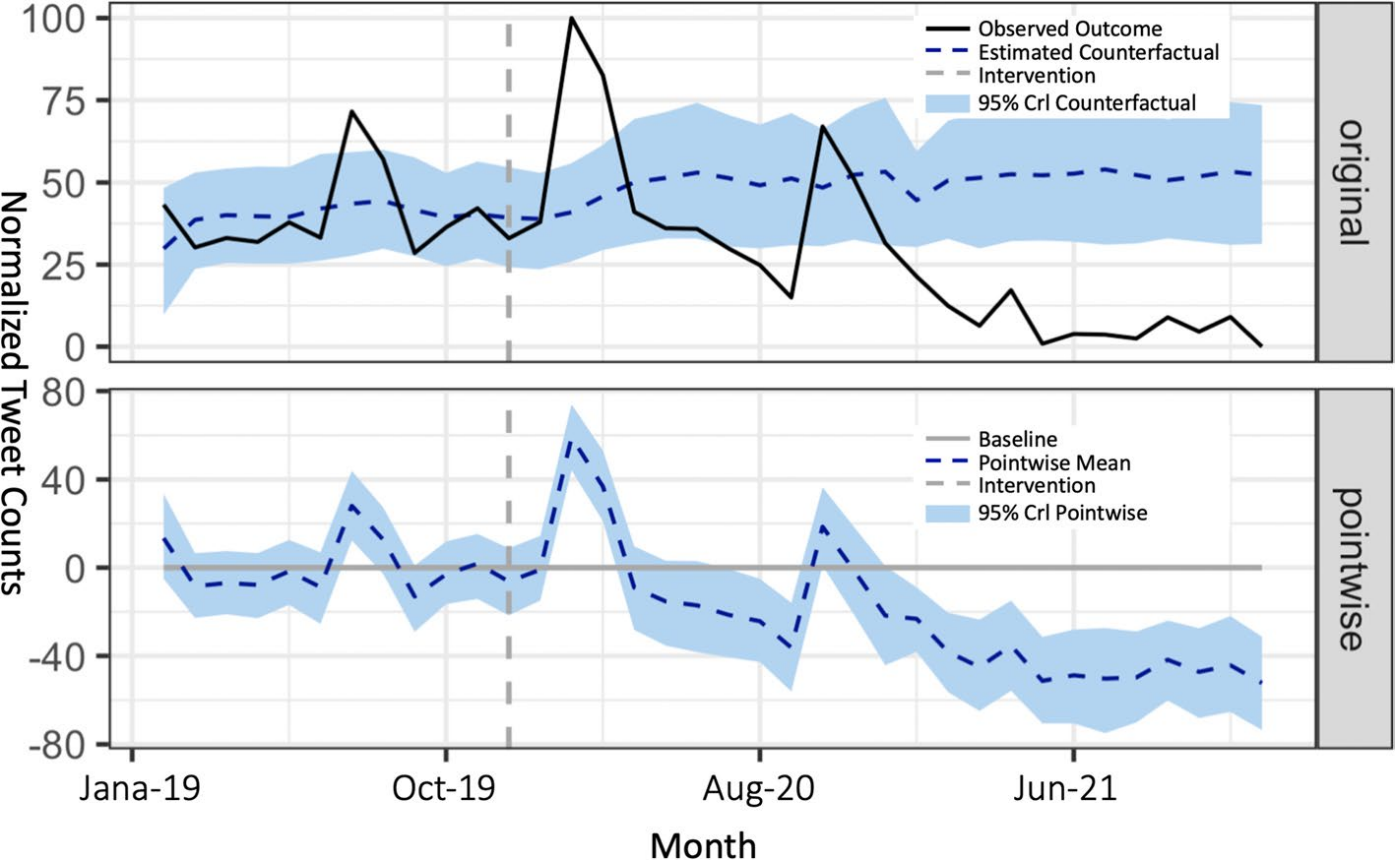
Causal impact analysis of COVID-19 on grocery-related discussions

We ran the causal impact analysis of the pandemic on the monthly counts of grocery-related tweets. The analysis reports a posterior tail-area probability *p*-value of 0.001, and the effect of the intervention, defined as the first case of COVID-19 reported in the US, is therefore significant. In Fig. 4, the vertical dotted line in month 12, i.e., March 20220, represents the intervention. The area before the line is span1 when the grocery-related discussions were not affected by the pandemic, and the area after the line is span2 and span3 after the pandemic began. The plot contains two panels.

The top panel shows the data and a counterfactual prediction for the post-treatment period. The solid black line is the actual observations of the normalized monthly tweet counts. The horizontal dotted line is the prediction of the normalized tweet counts based on the control series of the weather-related tweets, and it shows what the outcome would have looked like if the pandemic had never occurred. During the post-intervention period, the average of normalized online grocery-related tweet counts is approx. 24.40. By contrast, in the absence of intervention of COVID-19, we would have expected an average response of 40.83. The 95% interval of this counterfactual prediction is [37.03, 44.75]. The bottom panel shows the difference between observed data and counterfactual predictions. This is the pointwise causal effect, as estimated by the model. Subtracting this prediction from the observed response yields an estimate of the causal effect the intervention had on the normalized online grocery-related tweet counts. This pointwise causal effect is − 16.43 with a 95% interval of [− 20.36, − 12.63].

There were peaks on March 20 and November 20, 2021, demonstrating that people care more about groceries around these two times. However, their concerns about groceries have dropped considerably since then. The response variable showed a decrease of 40%. The 95% interval of this percentage was [− 50%, − 31%], and the negative causal effect can be considered statistically significant (Bayesian one-sided tail-area probability *p*-value = 0.001).

**Fig. 5:**
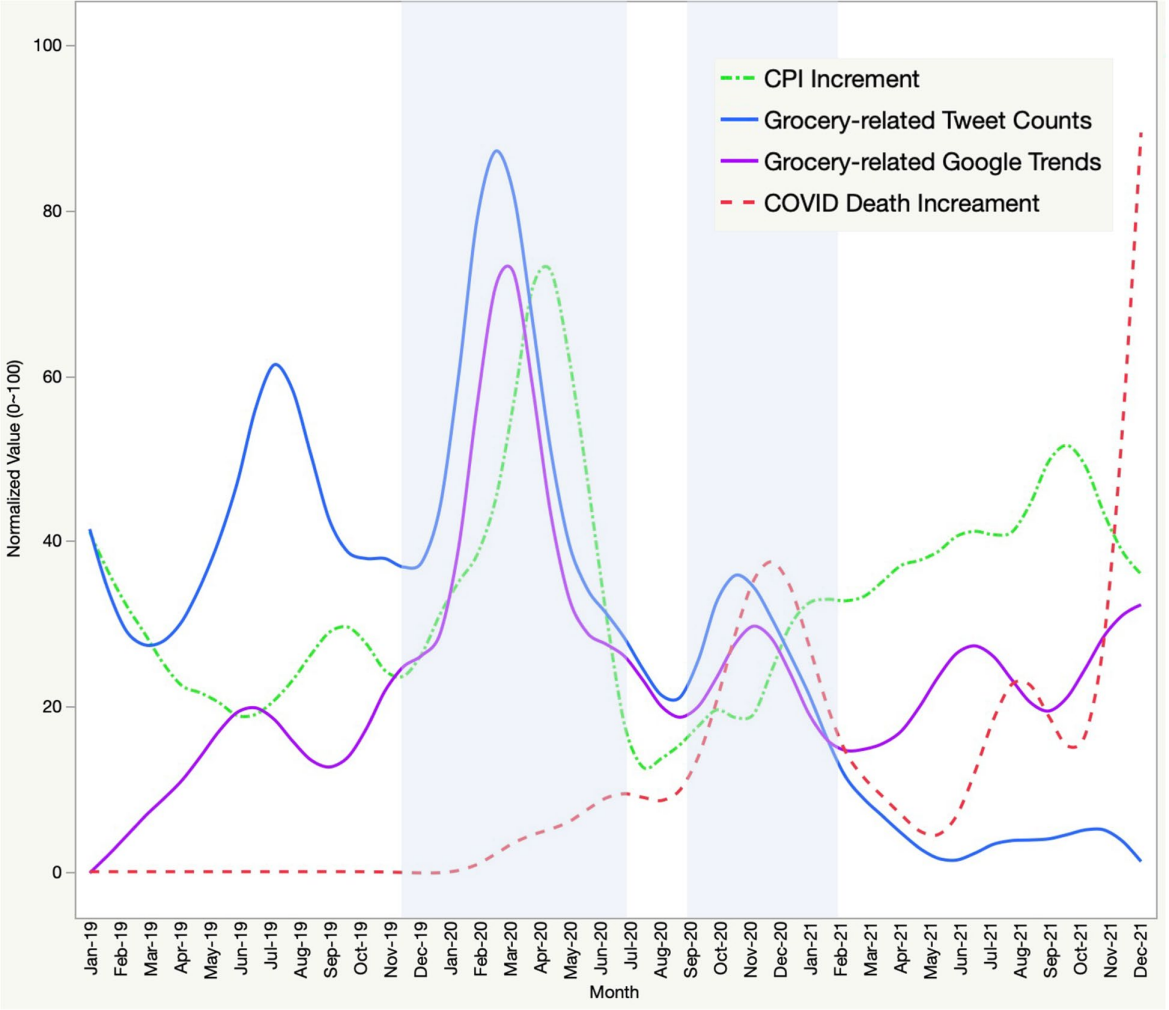
Trend analysis of grocery-related Tweet counts and Google Trends

Regarding online grocery shopping behaviors, we adopted grocery-related Google Shopping interest data on Google Shopping Trends as an overall indicator. In Fig. 5, we chose tweet counts and Google Shopping Trends interest index to represent the grocery shopping discussions and online shopping behaviors. The daily increase in COVID-19 death cases and CPI for food at home were presented as backdrops. It reveals that around March 11, 2020, when WHO declared COVID-19 a global pandemic (WHO, 2020), three curves reached their peaks: grocery-related tweet counts, Google Shopping Trends about online grocery shopping, and CPI increment for food at home. At first, the CPI increased, and a noticeable increase in grocery shopping intentions and online grocery shopping behaviors followed, corresponding to the grocery stock shortage and the soaring online orders around the same time. At the end of 2021, the daily COVID-death cases per day surged and triggered an increment in grocery shopping concerns and online grocery shopping behaviors. However, compared to the last peak, there were fewer reactions this time. Since then, people still made online grocery purchases, but the number of discussions on Twitter kept dropping.

## Discussion

### Main findings

People mainly discussed grocery shopping on weekend afternoons in the year before COVID-19. However, as the pandemic arose and progressed, the discussion of grocery shopping became more pervasive throughout the week. A year after the pandemic began, people had concerns about grocery shopping every day of the week. The pandemic is gradually changing people’s grocery shopping-related concerns and discussions.

Many studies have revealed that overwhelming exposure to COVID-19 can lead to depression and fear in the general public (Bao et al., 2020; Shigemura et al., 2020). During the long-term coexistence of the pandemic, fatigue also started to appear (Bodas & Peleg, 2021; Teng et al., 2020; WHO, 2020). Our findings assessed the public attitudes under the causal effect of COVID-19. The results revealed that people first experienced pandemic panic and then pandemic fatigue a year later when engaging in online grocery discussions and shopping. The variation in the quantity of grocery-related tweets also reflected geographic diversity in grocery concerns. States with small populations were more vulnerable to both pandemic panic and pandemic fatigue, while the populous states were more resilient to the impacts of pandemics. People in non-farm areas with less population and relatively lower levels of educational attainment tend to act more sensitively to the evolution of the pandemic.

There were two noticeable peaks when looking at the grocery-related discussions and online grocery shopping behaviors in relation to the COVID-19 death growth and CPI increment. In March 2020, when the virus was considered a public health emergency in the US, it caused panic buying. Grocery stock experienced a shortage, online orders soared, and the CPI for food at home increased most rapidly. In the second peak, when the COVID-19 death cases surged dramatically, people still talked about groceries on Twitter and made online grocery purchases, but the magnitude of reactions was significantly lower than before. When it came to 2021, people were still shopping online, which was in line with earlier studies that food e-commerce channels are likely to be associated with panic stockpile behaviors (Hao et al., 2020). Nonetheless, the public felt bored or paralyzed when it came to discussing grocery-related topics on Twitter and therefore, the topic’s popularity has been steadily dwindling.

### Limitations

With open access to Twitter big data, researchers can get the complete historical tweets to analyze the topic trends on Twitter using the full-archive search endpoint without any expense. However, we also need to acknowledge the sampling bias in geotagged tweets. In general, social media users tend to be younger, better educated and have higher incomes than US adults overall. (Barberá & Rivero, 2015). And the Twitter data is a non-uniform sample which is insufficient to represent the entire population (Mislove et al., 2011). It is recommended to quantify the degree of agreement between the sample distribution and population to evaluate the sampling bias for further exploration (Zhang & Zhu, 2018). In addition, even though geotagging on Twitter is regarded as a crucial proxy for understanding people’s thoughts and behaviors, the current research is limited to geotagged tweets, which only account for less than 3% of total tweets (Huang & Carley, 2019). To further reveal spatial and demographic information from social media, researchers advocated combining the location inference techniques based on user profiles and social networks (Davis Jr. et al., 2011; Huang & Carley, 2019; Tian et al., 2020).

Another limitation is that Google Shopping Trends data, although indicating the overall pattern and relative comparison, might not be a perfect mirror of search activity (Rovetta, 2021). The research could be more detailed with the actual online grocery shopping data with high spatial and temporal granularity. Similarly, if we had more precise CPI data at the state level, it would help demonstrate the geographic variation.

## Conclusions

COVID-19 has changed people’s shopping behaviors. Before the pandemic, people were used to discussing groceries on Twitter on weekend afternoons. During the pandemic, they started to talk about grocery-related topics during the daytime throughout the whole week. The findings also suggest that discussions on social media platforms such as Twitter could reflect pandemic panic and help detect pandemic fatigue during the pandemic. Both grocery-related discussions and online grocery shopping behaviors surged at the beginning of the pandemic. Nonetheless, due to the prolonged stress caused by covid, the public is increasingly reluctant to talk about the grocery. People first experienced panic buying groceries followed by pandemic fatigue a year later. The normalized tweet counts show a decrease of − 40% since the pandemic began, and the negative causal effect can be considered statistically significant (*p*-value = 0.001). To help reduce the anxiety towards pandemics and prevent the CPI for food at home from rapid growth, decision makers need to pay more attention to non-farm areas with less population. Policy or promotion aiming to maintain and reinvigorate public support for protective behaviors towards COVID-19 is recommended to target more on the population with lower education levels.

## Data Availability

The data that support the findings of this study are available from Twitter API v2 for Academic Research and Google Trends, but restrictions apply to the availability of these data, which were used under license for the current study, and so are not publicly available.
